# Efficacy of Neoadjuvant Short-Course Radiation Therapy Followed by Oxaliplatin-Based Chemotherapy for Locally Advanced Rectal Adenocarcinoma: A Single-Center Experience From Saudi Arabia

**DOI:** 10.7759/cureus.77604

**Published:** 2025-01-17

**Authors:** Tareq Salah, Mohamed Aboziada, Taleb Buhlaiaqh, Nada A Mass, Nedal Bukhari, Bader Alwhaibi, Abdossalam M Makhali, Mervat Mahrous, Sherif Mohamed, Nashwa Abd El-Aziz, Hoda Mokhtar

**Affiliations:** 1 Oncology Department, Prince Sultan Military Medical City, Riyadh, SAU; 2 Clinical Oncology and Nuclear Medicine Department, Faculty of Medicine, Assiut University, Asyut, EGY; 3 Radiation Oncology Department, South Egypt Cancer Institute, Asyut, EGY; 4 Medicine Department, King Saud University, Riyadh, SAU; 5 Oncology Department, Faculty of Medicine, Minia University, Minia, EGY; 6 Internal Medicine Department, Sultan Bin Abdulaziz Humanitarian City, Riyadh, SAU; 7 Medical Oncology Department, South Egypt Cancer Institute, Asyut, EGY; 8 Medical Oncology Department, Blood and Cancer Center, Riyadh, SAU; 9 Clinical Oncology Department, Faculty of Medicine, Minia University, Minia, EGY

**Keywords:** ksa: kingdom of saudi arabia, locally advanced rectal cancer, neoadjuvant, oxaliplatin, radiation oncology, short course, systemic chemotherapy

## Abstract

Background

The 5-fluorouracil (5-FU), capecitabine-based long-course or short-course radiotherapy (SCRT) eventually preceded or followed by induction or consolidation chemotherapy (CT) and resection represents the preferred regimen for the treatment of locally advanced rectal cancer (LARC). This study aims to report our experience as a large medical center in Saudi Arabia, with the efficacy of short-course radiation therapy followed by oxaliplatin-based CT in achieving a pathologic complete response (pCR) in patients with LARC.

Materials and methods

This retrospective analysis encompassed 57 patients diagnosed with LARC at a large tertiary center in Riyadh, Saudi Arabia, from June 2020 to December 2022. All participants underwent short-term radiotherapy (25 Grays (Gy) over fractions within one week) followed by CT with 5-FU, leucovorin, and oxaliplatin (FOLFOX) or capecitabine and oxaliplatin (CAPOX), constituting the total neoadjuvant therapy (TNT). Surgical intervention and total mesorectal excision were performed six to eight weeks post-preoperative treatment. The primary endpoint was the pCR rate.

Results

Of the study participants, 34 (60%) were males, with a mean age of 57.6 ± 13.9 years. Two-thirds (n = 37,65%) were classified as T3. The overall response rates were 12 (21%), 12 (21%), 24 (42%), and nine (16%), for complete response (CR), near-complete response (nCR), partial response (PR), and progressive disease (PD), respectively. The multivariable logistic regression model identified five independent predictors for overall CR after adjusting for disease-related factors: N-stage, the circumferential resection margin (CRM), average vascularity (AV), surgical procedure, and postoperative tumor size. Patients with N2 disease had an 18% lower chance of achieving CR (OR = 0.824; 95% CI: 0.634-0.974; p = 0.035). Positive CRM was linked to a 71% reduction in the probability of CR (OR = 0.268; 95% CI: 0.087-0.823; p = 0.021). Each 1 cm increase in AV corresponded to a 28.5% increase in the likelihood of complete response (OR = 1.285; 95% CI: 1.029-1.605; p = 0.027). Patients who underwent AR had 2.8 times greater chances of achieving CR than those who underwent abdominoperineal resection (APR) (OR = 2.801; 95% CI: 1.057-9.324; p = 0.044). Lastly, each 1 cm increase in postoperative tumor size was associated with a 92.5% reduction in the odds of CR (OR = 0.074; 95% CI: 0.017-0.330; p = 0.001).

Conclusions

The current study supports the efficacy of TNT for treating LARC, with a pCR rate of 21% and near-complete response in nearly half of the patients with LARC. Significant predictors of pCR included N-stage, CRM status, AV size, and surgical approach. These insights could refine patient selection for TNT and inform future strategies to optimize treatment outcomes in rectal cancer. Prospective multicenter studies are warranted.

## Introduction

Neoadjuvant conventionally fractionated chemoradiation (CTRT) with a 6-8 week or longer interval from surgery is the standard treatment for patients with locally advanced or unresectable rectal cancer (LARC). Despite this, survival rates are still unsatisfactory, and many patients relapse either locally or systemically, leading to five-year local and distant relapse- and disease-free survival (DFS) rates of 10%-12%, 20%-25%, and about 70%, respectively [1]. While early studies have shown promise and robust evidence on the efficacy of short-course radiotherapy (SCRT) followed by neoadjuvant chemotherapy (CT), the five-year follow-up of rectal cancer and preoperative induction therapy followed by dedicated operation (RAPIDO) trial showed that treatment was associated with an increased risk of locoregional relapse (LRF)[2]. Various trials have added oxaliplatin to CTRT regimens with an improvement in pathologic complete response (pCR), but this benefit did not translate into an improvement in overall survival (OS) [1].

Including CT intensification before or after CTRT (termed the total neoadjuvant therapy, or TNT, strategy) may increase the number of negative margin resections, potentially reduce distant metastases, and possibly prolong OS [3]. Thus, the 5-fluorouracil (5-FU), capecitabine-based, long-course or short-course radiotherapy (SCRT), eventually preceded or followed by induction or consolidation CT and resection, now comprise the preferred regimen for the treatment of locally advanced disease. 

This study's objective is to assess the efficacy of short-course radiation therapy followed by oxaliplatin-based CT in achieving a pCR in patients with locally advanced rectal adenocarcinoma at a large medical center in Saudi Arabia.

## Materials and methods

Study design and population

This investigation is a retrospective analysis that encompassed 57 patients diagnosed with histopathology-confirmed middle and distal locally advanced rectal adenocarcinoma (cT2, cT3, or cT4, N0-N2, M0) through magnetic resonance imaging (MRI) based on the American Joint Committee on Cancer (AJCC) eighth Edition criteria [4], from June 2020 to December 2022. The study protocol received ethical clearance from the local ethics committee of the Prince Sultan Military Medical City (PSMMC), Riyadh, Saudi Arabia (protocol reference number 1061).

Treatments

All participants underwent short-term radiotherapy (5 × 5 Gy over fractions within one week) followed by nine cycles of CT with 5-FU, leucovorin, and oxaliplatin (FOLFOX) or six cycles of capecitabine and oxaliplatin (CAPOX) constituting the TNT. Restaging was performed one to two weeks after the last CT cycle by computed tomography of the thorax, abdomen, and pelvis and MRI of the pelvis. An additional MRI of the pelvis was recommended in the middle of the neoadjuvant CT to disclose any signs of progression [4]. Surgical intervention and total mesorectal excision were performed six to eight weeks post-preoperative treatment.

Assessment and outcomes

Treatment response was assessed after neoadjuvant treatment (based on baseline and restaging MRI reports) and after surgery (based on pathology reports). For this report, all patients with a decrease in T-stage and/or N-stage compared with baseline MRI stage were defined as good responders (i.e., downstaging was accomplished). Follow-up was accomplished according to a standardized protocol. Outpatient visits were scheduled at 6, 12, 24, 36, and 60 months after surgery. Patients who were lost to follow-up, withdrew informed consent, or died before surgery were excluded from the study. The primary endpoint of this study was the pCR rate, defined as the absence of any residual tumor cells in the primary tumor and lymph nodes (ypT0N0) [5-7].

Statistical analysis

Data verification, coding, and analysis were conducted using IBM SPSS Statistics for Windows, Version 24 (Released 2016; IBM Corp., Armonk, New York, United States). Descriptive statistics encompassed means, standard errors, medians, interquartile range (IQR), and percentages. Chi-square tests were employed to compare frequency distributions across different groups. Normality tests were performed using the Shapiro-Wilk/Kolmogorov-Smirnov method for continuous variables. For continuous variables with multiple categories, analysis of variance (ANOVA) was utilized to compare mean differences among groups. To assess independent predictors for complete response, multivariable logistic regression analysis was performed. A significance threshold was set at p < 0.05.

## Results

Baseline characteristics of the participants

Fifty-seven patients were included in this study; 34 (60%) were males, with a mean age of 57.6 ± 13.9 years. Two-thirds (n = 37, 65%), nine (16%), and three (5%) were classified as T3, T4, and T2, respectively. The average time to surgery was 8.7 ± 1.8 weeks, with anterior resection (AR) performed in two-thirds of cases (n = 38) and abdominoperineal resection (APR) in 33.3% (n = 19). The median postoperative tumor size was 2 cm. Positive nodes were identified in 14 (24.6%) patients with a mean size of 2.7 ± 4.2 cm. The overall response rates were 12 (21%), 12 (21%), 24 (42%), and nine (16%), for complete response (CR), near-complete response (nCR), partial response (PR), and progressive disease (PD), respectively (Table 1).

**Table 1:** Clinical and pathological baseline characteristics of the enrolled subjects (n = 57) CRM: circumferential resection margin; AR: anterior resection; APR: abdominoperineal resection; IQR: interquartile range; SD: standard deviation

Item	Number (%)
Age (years)	
Mean ± SD	57.56 ± 13.9
Median (IQR)	57 (15)
Gender	
Male	34 (59.6%)
Female	23 (40.4%)
T-stage	
T2	3 (5.3%)
T3	37 (64.9%)
T4	9 (15.8%)
T4a	4 (7%)
T4b	4 (7%)
N-stage	
N0	7 (12.3%)
N1	20 (35.1%)
N2	30 (52.6%)
Size of the lesion (centimeter)	
Mean ± SD	5.49 ± 1.9
Median (range)	5 (2)
CRM	
Negative (>1 mm)	30 (52.6%)
Positive (≤1 mm)	27 (47.4%)
Distance from the anal verge (AV) (centimeter)	
Mean ± SD	5.80 ± 2.7
Median (range)	6 (4)
Surgical procedure	
AR	38 (66.7%)
APR	19 (33.3%)
Postoperative tumor size (centimeter)	
Mean ± SD	1.79 ± 1.5
Median (range)	2 (2)
Positive node (yes)	14 (24.6%)
Overall response	
Complete response	12 (21.1%)
Near-complete response	12 (21.1%)
Partial response	24 (42.1%)
Progressive disease	9 (15.7%)

Correlates of overall response

The rates of partial and complete responses were significantly higher among T2/T3 cases (70.8% and 79.2%) compared to those with T4 (29.2% and 20.8%) (p = 0.049), respectively. Response rates were significantly associated with N-staging (p = 0.039), indicating that better responses correlated with lower staging. Positive CRM was significantly more in patients with PD (n = 7, 77.8%) than in those with partial or complete responses (n = 13, 54.2%; n = 7, 29.2%) (p = 0.011), respectively. A positive correlation was observed between mean average vascularity (AV) size and response improvement (p = 0.035).

The mean postoperative tumor size was significantly smaller in patients with CR (0.4 ± 0.1 cm) compared to those with PD (2.6 ± 0.3 cm, p < 0.001) and PR (2.9 ± 0.4 cm), p < 0.001, respectively. The positive lymph nodes (LNs) were significantly higher in patients with PD (5 out of 9, 55.6%) than in those with PR (9 out of 24, 37.5%) and 0 (0%) in those with CR (p < 0.001), respectively.

The mean number of actual LN was significantly higher in patients with PD (15.2 ± 4.40) compared to those with PR (12.3 ± 3.9), and CR (12.2 ± 4.2 cm) (p = 0.047), respectively. There was no significant correlation between overall response on the one hand and age, gender, size of the lesion, dose of RT, and type of surgical procedure on the other hand (Table 2).

**Table 2:** Correlates of overall response T: tumor; N: node; Gy: grays; CRM: circumferential resection margin; AR: anterior resection; APR: abdominoperineal resection; cm: centimeter; LN: lymph node; ANOVA: analysis of variance *ANOVA test was used to compare the mean difference between groups. **A post hoc test with Bonferroni corrections was used to compare the mean difference between groups. ***The Chi-square test was used to compare the frequency between groups. ^§^number ± standard deviation. ^¶^Test statistics value: t-score for t-test, the Chi-square value for the Chi-square test, and f-value for ANOVA test

Variable	Overall response (n=57)			P- value	Test statistics^¶^
	Progressive disease (n = 9)	Partial response (n = 24)	Complete response (n = 24)		
Age^§^ (years)	55.44 ± 14.5	59.75 ± 15.4	56.17 ± 12.2	0.559*	0.599
Gender					
Male	3 (33.3%)	17 (70.8%)	14 (58.3%)	0.182***	3.854
Female	6 (66.7%)	7 (29.2%)	10 (41.7%)		
T-stage					
T2/3	4 (44.4%)	17 (70.8%)	19 (79.2%)	0.049***	4.157
T4	5 (55.6%)	7 (29.2%)	5 (20.8%)		
N-stage					
N0	1 (11.1%)	0 (0%)	6 (25%)	0.039***	6.019
N1	1 (11.1%)	12 (50%)	7 (29.2%)		
N2	7 (77.8%)	12 (50%)	11 (45.8%)		
Size of the lesion (cm)	6.04 ± 1.9	5.70 ± 1.4	5.07 ± 1.2	0.355*	0.355
CRM					
Negative	2 (22.2%)	11 (45.8%)	17 (70.8%)	0.011***	6.973
Positive	7 (77.8%)	13 (54.2%)	7 (29.2%)		
Average vascularity size	4.22 ± 0.9	5.40 ± 0.6	6.80 ± 0.8	0.035*	3.563
Dose GY	52.78 ± 2.6	52.67 ± 2.5	52.71 ± 2.7	0.994*	0.006
Surgical procedure					
APR	3 (33.3%)	11 (45.2%)	5 (20.8%)	0.193***	3.375
AR	6 (66.7%)	13 (54.8%)	19 (79.2%)		
Postoperative tumor size (cm)	2.56 ± 0.3	2.90 ± 0.4	0.0	<0.001*	21.750
Postoperative positive LN	5 (55.6%)	9 (37.5%)	0 (0%)	<0.001***	14.650
Number of actual LN	15.22 ± 4.4	12.29 ± 3.9	12.21 ± 4.2	0.047*	4.617

Predictors of complete response

We used the multivariable logistic regression model to identify the predictors of complete overall response. Five independent predictors were identified after adjusting for disease-related factors: N-stage, CRM, AV, surgical procedure, and postoperative tumor size. Patients with N2 disease had an 18% lower chance of achieving CR (OR = 0.824; 95% CI: 0.634-0.974; p = 0.035). Positive CRM was linked to a 71% reduction in the probability of CR (OR = 0.268; 95% CI: 0.087-0.823; p = 0.021). Each 1 cm increase in AV corresponded to a 28.5% increase in the likelihood of complete response (OR = 1.285; 95% CI: 1.029-1.605; p = 0.027). Patients who underwent AR had 2.8 times greater chances of achieving CR compared to those who underwent APR (OR = 2.801; 95% CI: 1.057-9.324; p = 0.044). Lastly, each 1 cm increase in postoperative tumor size was associated with a 92.5% reduction in the odds of CR (OR = 0.074; 95% CI: 0.017-0.330; p = 0.001) (Table 3). 

**Table 3:** Predictors of complete response: multivariable regression analysis OR: odds ratio; CI: confidence interval; AV: average vascularity; CRM: circumferential resection margin; PO: postoperative; AR: anterior resection

Variable	OR (95% CI)	p-value
Age (years)	1.014 (0.948-1.026)	0.287
Gender (male)	1.183 (0.396-3.536)	0.563
N-stage (N2)	0.824 (0.634-0.947)	0.035
Positive CRM	0.268 (0.087-0.823)	0.021
AV (cm)	1.285 (1.029-1.605)	0.027
Surgical procedure (AR)	2.801 (1.057-9.324)	0.044
PO tumor size (cm)	0.074 (0.017-0.330)	0.001

## Discussion

Colorectal cancer ranks as the most prevalent cancer among Saudi males and the third among females, making it the second most common overall [8]. SCRT and long-course chemoradiotherapy (LCCRT) exhibit comparable rates of locoregional relapse, DFS, and OS for each radiation regimen. Despite effective pelvic control, the risk of distant metastasis remains around 25% to 30% within three to five years, highlighting the necessity for innovative strategies to diminish this risk. One approach involves intensifying preoperative treatment through the use of TNT, integrating both radiation and systemic therapy before surgery [9,10]. Although SCRT followed by CT presents as a compelling alternative to traditional long-course CRT, the efficacy of this approach in achieving pCR and enhancing patient outcomes remains inadequately understood.

Analysis comparing TNT (CAO/ARO/AIO-12) to intensified neoadjuvant and adjuvant treatment (CAO/ARO/AIO-04) indicates that TNT enhances rates of pathological complete remission in comparison to intensified neoadjuvant LCCRT but does not enhance oncological outcomes [11]. The current study aimed to evaluate the efficacy of SCRT followed by oxaliplatin-based CT (FOLFOX or CAPOX) as a part of TNT in patients with LARC. The findings revealed that the overall response rates were 12 (21%), 12 (21%), 24 (42%), and nine (16%) for CR, nCR, PR, and PD, respectively.

These results align with anticipated outcomes from TNT strategies in LARC, where the combined modality aims to reduce tumor burden significantly. [12] A noteworthy 16% of patients exhibited progressive disease (PD), which may indicate inherent resistance to the neoadjuvant therapy regimen. Our analysis showed no significant correlation between overall response and demographic factors such as age or gender, which agrees with previous studies. [13]. However, a significantly better response was observed in patients with lower T-stages (T2/T3 vs. T4) and lower N-stages (N0/N1 vs. N2), reinforcing evidence that advanced disease correlates with diminished response to neoadjuvant therapy. [9,14]

Our current ability to predict CRT response in patients with rectal cancer remains limited. [15] The current study identified several significant factors related to treatment response, including clinical T-stage, N-stage, and circumferential resection margin (CRM). These observations corroborate earlier research indicating that improved responses are typically associated with lower T-stage N-stage and negative CRMs. These findings emphasize the necessity of achieving negative CRMs in preoperative evaluations and the need for aggressive treatment strategies in patients with positive margins.

Interestingly, the tumor's lesion size and AV also influenced treatment response. While lesion size did not significantly correlate with overall response, a trend suggested that larger lesions may be associated with poorer responses. This may reflect the principle that larger tumors often exhibit more aggressive biology, reducing the likelihood of full therapeutic response. More intriguingly, the AV, which indicates tumor vascularity, was significantly correlated with improved treatment outcomes, possibly related to enhanced drug delivery and efficacy in well-vascularized tissues. [16] Another significant finding of the current study was the notable correlation between the type of surgical procedure and treatment response. Patients who underwent AR were significantly more likely to achieve a CR, which may relate to the overall lower tumor burden, potentially superior surgical outcomes for patients with more localized disease, and the capacity for more precise resections [17].

Multivariable logistic regression analysis identified several independent predictors of CR. The odds of achieving a CR decreased by 18% for each increment of N2 disease and by 71% for positive CRM. Conversely, larger AV and AR surgery were associated with an increased likelihood of CR, with patients undergoing AR surgery having 2.8 times the odds of achieving CR. These findings support the notion that tumor biology, surgical approach, and margin status are critical determinants of treatment success [1,18].

Compared to other large-scale studies, such as the RAPIDO trial [19], which assessed SCRT followed by CT, our results demonstrate a comparable trend in treatment efficacy, with pCR rates around 20%. However, variations in outcomes may stem from differences in patient selection, CT regimens, and radiation protocols. For instance, in the UNICANCER-PRODIGE 23 trial [20], the intensification of CT (e.g., incorporating irinotecan with FOLFOX) alongside SCRT yielded promising results concerning survival and pCR rates. Our study, which utilized only oxaliplatin-based regimens, suggests that although these regimens remain effective, further benefit may be derived from intensification [21].

Overall, our results agree with those of the recent meta-analysis [1], which indicated that 5-FU, capecitabine-based (long course) CTRT, or SCRT, eventually preceded or followed by induction or consolidation CT and resection, now comprise the preferred regimen for the treatment of LARC.

Limitations

This study does have limitations. The retrospective design and single-center nature may restrict the generalizability of the findings. The small sample size could also diminish statistical power, especially when evaluating factors with moderate effect sizes. Furthermore, the study did not assess long-term outcomes such as OS and DFS, which would provide a more comprehensive evaluation of treatment efficacy.

## Conclusions

The current study supports the effectiveness of TNT for treating LARC, with a pCR rate of 21% and nCR in nearly half of the patients. Significant predictors of CR included N-stage, CRM status, AV size, and surgical approach. These insights could refine patient selection for TNT and inform future strategies to optimize the treatment outcomes in rectal cancer. Future research should focus on validating these findings in larger, multicenter cohorts and exploring molecular and genetic factors that may influence treatment responses in this patient population.
